# Climate change-induced increases in precipitation are reducing the potential for solar ultraviolet radiation to inactivate pathogens in surface waters

**DOI:** 10.1038/s41598-017-13392-2

**Published:** 2017-10-12

**Authors:** Craig E. Williamson, Sasha Madronich, Aparna Lal, Richard G. Zepp, Robyn M. Lucas, Erin P. Overholt, Kevin C. Rose, S. Geoffrey Schladow, Julia Lee-Taylor

**Affiliations:** 1 Department of Biology, Miami University, Oxford, Ohio, 45056 USA; 2 National Center for Atmospheric Research, Boulder, Colorado, 80307 USA; 3 National Centre for Epidemiology and Population Health, The Australian National University, Canberra, Australian Capital Territory 2600 Australia; 4 United States Environmental Protection Agency, Athens, Georgia 30605 USA; 5 Department of Biological Sciences, Rensselaer Polytechnic Institute, Troy, New York, 12180 USA; 6 Department of Civil and Environmental Engineering, University of California, Davis, California USA

## Abstract

Climate change is accelerating the release of dissolved organic matter (DOM) to inland and coastal waters through increases in precipitation, thawing of permafrost, and changes in vegetation. Our modeling approach suggests that the selective absorption of ultraviolet radiation (UV) by DOM decreases the valuable ecosystem service wherein sunlight inactivates waterborne pathogens. Here we highlight the sensitivity of waterborne pathogens of humans and wildlife to solar UV, and use the DNA action spectrum to model how differences in water transparency and incident sunlight alter the ability of UV to inactivate waterborne pathogens. A case study demonstrates how heavy precipitation events can reduce the solar inactivation potential in Lake Michigan, which provides drinking water to over 10 million people. These data suggest that widespread increases in DOM and consequent browning of surface waters reduce the potential for solar UV inactivation of pathogens, and increase exposure to infectious diseases in humans and wildlife.

## Introduction

Climate change is profoundly altering ecosystems and the goods and services that they provide^1^. While warming temperatures have been the central focus of studies on climate change from genes to ecosystems^2^, increases in extreme precipitation events (droughts and floods) are also rendering fundamental changes in aquatic ecosystems ranging from inland lakes^3^ to the Gulf of Mexico^4^ and the Great Barrier Reef^5^. Outbreaks of waterborne infectious diseases are often associated with heavy precipitation events^6^. Aquatic ecosystems are hotbeds of disease transmission because pathogens can avoid desiccation, and hosts abound. Waterborne pathogens of humans and wildlife include infectious viruses, bacteria, protozoans, and fungi. In the United States alone, between 12 and 19 million people annually contract infectious waterborne diseases^6^. Epidemics in wildlife species can alter food webs, community composition, genetic diversity, and biogeochemical cycling^7^.

Control of waterborne pathogens is complex, has been extensively investigated for a wide variety of pathogens, and models have been developed to estimate the impact of the multiple types of environmental control^8–10^. Among these controls, solar ultraviolet radiation (UV) is widely recognized as a significant factor inactivating pathogens in the natural environment^10–12^. Here we explore a previously suggested but largely unrecognized mechanism through which heavy precipitation may increase waterborne pathogens. This mechanism involves increases in terrestrially-derived DOM that reduce penetration of solar UV in surface waters^13^. DOM strongly and selectively absorbs the shorter wavelength UV in sunlight, thus reducing the potential for solar UV to inactivate waterborne pathogens^14,15^. Although the ability of solar UV to inactivate waterborne pathogens has long been recognized, the importance of this nexus of precipitation and DOM-mediated reductions in water transparency and solar UV inactivation of pathogens has not previously been quantified. Here we examine the evidence for this link by first highlighting recent literature on the sensitivity of waterborne pathogens to the UV in natural sunlight. We then quantitatively assess the potential for natural variations in incident sunlight and water transparency to alter inactivation of various pathogens. We present a case study from Lake Michigan that demonstrates the effects of storm events on the potential for solar disinfection, and discuss the implications of changes in UV exposure in inland and coastal waters for waterborne pathogens and the spread of infectious disease globally.

## Waterborne pathogens are inactivated by solar UV radiation

The potential for solar radiation to inactivate pathogens in drinking water in various types of UV-transparent containers (SODIS) is well established and recently has been reviewed^16,17^. The ability of solar UV-B radiation to inactivate bacteria in natural oceanic waters and the reduction of this solar inactivation potential in waters with reduced transparency has also been documented^18^. Collectively these data provide strong evidence for the potential of sunlight to inactivate pathogens in natural surface waters including coastal and inland waters on which humans and wildlife are heavily dependent.

Pathogenic viruses and bacteria are generally susceptible to UV-induced inactivation, with significant inactivation by sunlight occurring in less than one day during summer for even the most resistant viruses^19^. Full sunlight can induce a ten-fold (log_10_) inactivation within a relatively short time, e.g. 2 h in sunlight vs. 9–53 h in the shade for *Enterococcus* spp. and *Escherichia coli*, two important groups of bacteria used as faecal indicators of warm-blooded animals including humans^20^. Experiments with these same bacteria in open wetlands showed 2–3 log_10_ reductions from the inlet to the outlet, but only 0.5–0.6 log_10_ reductions when floating-leaved plants covered the water surface and blocked sunlight^21^. Pigmented enterococci in this study did not show a significant decrease in response to sunlight exposure. *Campylobacter jejuni*, one of the most common causes of food poisoning in humans, showed a two-fold greater survival time to 90% inactivation (21.8 h) when incubated in the shade vs. the sunlight in an *in situ* incubation experiment in the summer^22^. In aquatic ecosystems, the sensitivity of cyanophages to UV-induced damage may reduce their ability to control harmful blooms of cyanobacteria^23^.

Some protozoan pathogens are also rapidly inactivated by sunlight. *Cryptosporidium parvum* is a common and widespread contaminant in surface waters and an indicator species for protozoan pathogens in drinking water. It causes cryptosporidiosis that can lead to chronic illness and even death in children, the elderly, and the immune-compromised. Infectious oocysts of *C*. *parvum* are spread primarily by faecal-oral contact by humans and livestock. The oocysts are resistant to chlorination, but natural solar UV substantially reduces (ten fold or greater) their *in vitro* infectivity^24^. Other experiments have shown up to a 90% inactivation within an hour for *C parvum* exposed to natural sunlight^25^. In this latter study, natural waters with higher DOM concentrations reduced this solar UV inactivation, with the extent of the inactivation depending on the source water and DOM concentration. Effective inactivation by sunlight has also been demonstrated in a variety of non-human waterborne pathogens including those that infect lizards^26^, Atlantic salmon^27^, birds^28^, and zooplankton^14^. The effects of UV on the chytrid fungal pathogen *Batrachochytrium dendrobatidis* (Bd), which has caused worldwide declines in amphibian populations, remains uncertain. While Bd zoospores are sensitive to natural sunlight, higher infection prevalence has been observed in ponds with higher water transparency^29^.

## Modeling the Solar Inactivation Potential (SIP) in nature

The UV spectrum can be divided into UV-A (315–400 nm), UV-B (280–315 nm) and UV-C (200–280 nm) wavebands. While germicidal lamps for disinfection use primarily the short wavelength, high energy UV-C radiation, here we focus on the inactivating effects of natural solar radiation. Ozone in the stratosphere prevents any UV-C from reaching Earth’s surface and removes most of the solar UV-B, particularly wavelengths shorter than about 295 nm. But the longer wavelength UV-B that reaches Earth’s surface is still potent enough to induce damage to DNA as well as other cellular components^30^.

Quantifying the effects of solar UV on pathogens in aquatic environments requires knowledge of (1) the number of photons and spectral composition (how many photons at each wavelength) of the UV reaching the surface of the water, (2) the transparency of the water to UV, including spectral absorption, and (3) the spectral sensitivity of the pathogen, referred to as an action spectrum or biological weighting function. The indirect effects of UV (e.g. by producing reactive oxygen species, ROS, such as singlet oxygen, especially in the UV-A region) may also play an important role in disinfection^15,31,32^. In some cases for some viruses such as MS2, these can be the primary means of disinfection^10^. However, these studies and others^33^ suggest that direct UV-induced damage to DNA by the shortest wavelengths in solar radiation is often the dominant process. The spectral sensitivity of viruses to inactivation by UV has been demonstrated to be very similar to the spectral sensitivity of DNA damage^11^. Here we use the DNA action spectrum to model the solar inactivation potential (SIP), defined as the DNA-weighted UV exposure (J m^−2^ day^−1^). For the underwater estimates for waterborne pathogens we average this over the top 1 meter depth since these surface waters are where humans, wildlife, and many aquatic organisms are likely to have the most exposure. We estimated SIP using the biologically active UV-B reaching the water surface and penetrating into the water using the Tropospheric Ultraviolet-Visible (TUV, version 5.3) model^34^. The sensitivity of different pathogens to UV-C in germicidal lamps has been more extensively investigated than that for solar UV-B. Therefore, as a first order estimate, we used available data on UV-C inactivation and the DNA action spectrum to estimate the SIP of several pathogens and indicator species for natural solar radiation (Table 1). The value of this modeling approach has been developed and explored for viruses and found to be well supported by experimental data^11^.

**Table 1:** Estimates of UV radiation levels necessary for inactivation of common human pathogens and related indicator species.

Target	Fluence Rate for a decade reduction at 254 nm^(a)^ (J m^−2^)	Number of 10-fold reductions day^−1^ for typical solar UV^(b)^	Notes^(c)^
Adenovirus 40	300	0.3	Hg^71^
Adenovirus 41	236	0.4	Hg^71^
Coliphage MS-2	140	0.7	Hg^71^
Coliphage PRD-1	87	1.1	Hg^71^
Poliovirus type 1	41	2.4	Hg^71^
Hepatitis A	40–92	1.1–2.5	Est.^72^
*Giardia lamblia*	<2.5	>4	Hg^73^
*Giardia lamblia*	<15	>7	Hg^74^
*Cryptosporidium parvum* (infectivity reduction)	5	20	Hg^75^

^(a)^90% inactivation fluence at 254 nm, reported by the cited sources. These are either direct measurements under a Hg lamp, or estimated from (among other factors) genome size. ^(b)^Number of 10-fold reductions during 24 hours (one day) under exposure to a typical solar tropospheric ultraviolet radiation amount equivalent to 100 J m^−2^ day^−1^ of 254 nm radiation (computed using the Setlow DNA action spectrum, normalized at 254 nm, to “translate” the damage from 254 nm to tropospheric wavelengths mostly contributing in the 295–315 nm region). ^(c)^Method (Hg = low pressure Hg lamp; Est. = estimated from genome model); number gives reference source.

**Figure 1:**
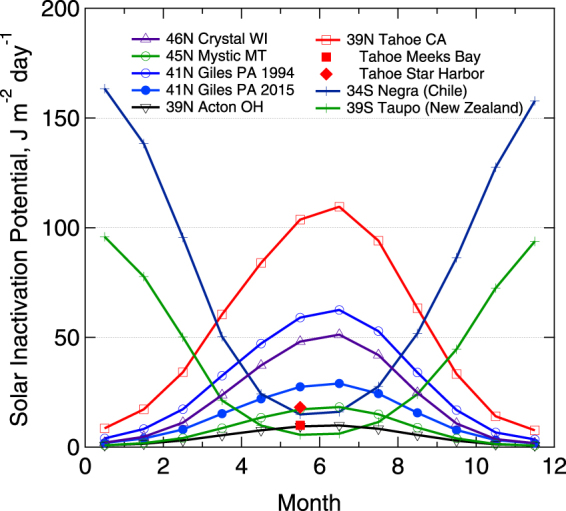
Seasonal and interannual variation in SIP in a suite of temperate lakes. SIP is averaged over the top one m depth, and varies primarily as a function of differences in seasonally incident UV, water transparency to UV, and to a lesser extent elevation (Tahoe is 1,897 m above sea level, and Negra, 2,700 m). UV transparency varies within lakes as well (e.g. lower in nearshore bays and harbors than offshore in Tahoe, and because of long-term browning in more recent decades in Giles). Note the inverse seasonal patterns in the Northern Hemisphere vs. Southern Hemisphere. Lake latitude, name, and U.S. state or country are given.

The clarity of inland waters and thus the potential for solar inactivation of waterborne pathogens varies greatly over space and time. For example, 1% of incident 320 nm UV reaches depths of tens of meters in some of the most transparent lakes and drinking water reservoirs in the world, but only a few centimeters in less transparent lakes^35^. DOM and other dissolved and particulate compounds reduce water transparency and thus exposure to disinfecting solar UV as well. Modelling the SIP for lakes in the Northern and Southern Hemispheres for which we have water transparency data reveal strong differences over space and time (Fig. 1). SIP can be reduced by an order of magnitude due to (1) increases in sun angle related to time of year, (2) the lower water transparency in some lakes vs. others, and (3) reduced water transparency in nearshore vs. offshore sites in Lake Tahoe (Fig. 1). The nearshore habitats of Lake Tahoe where SIP is low, are also where human use is high, and water intakes are located^36^, thus leading to potential increases in human exposure to pathogens.

## Changes in DOM alter SIP in surface waters

**Figure 2:**
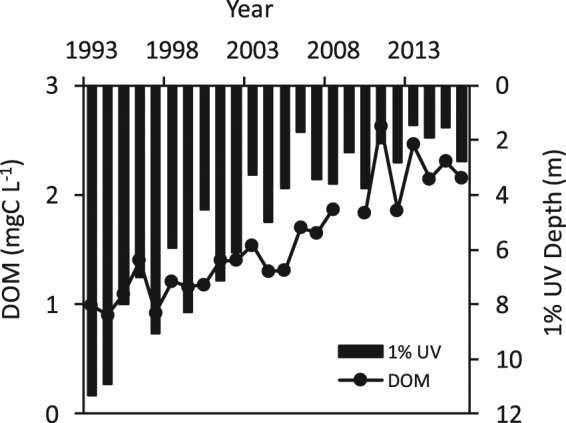
Browning-induced reductions in UV transparency in Lake Giles, northeastern Pennsylvania, USA. Long-term increases in DOM and decreases in UV transparency (depth to which 1% of subsurface 320 nm penetrates) related to browning of this and other lakes in the northeastern USA have corresponded closely with a two-fold or greater reduction in SIP (see Fig. 1).

One of the strongest signals of changing water clarity in inland waters in recent decades is the doubling of the concentrations of DOM in regions of northeastern North America and northern Europe^37^. This phenomenon, known as browning, can substantially reduce water clarity on time scales ranging from individual storm events to seasonal, inter-annual, and inter-decadal time periods^35^. The causes of browning are multiple and include recovery from acidification^37^, and increased precipitation related to climate change^35,38^. The increases in the quantity as well as darker color of terrestrially-derived DOM associated with browning result in greatly increased absorption of UV and reduce SIP, fostering increased survival of waterborne pathogens. For example, long-term increases in DOM and reductions in UV transparency in Lake Giles in northeastern North America^39^ have been very closely correlated over time in recent decades (Fig. 2), and translate to a two-fold reduction in SIP (Fig. 1).

The penetration of UV into water bodies is controlled in large part by input of terrestrially-derived DOM to surface waters^35,39^ and therefore by precipitation and hydrologic conditions^38^. Increases in precipitation lead to water-saturated wetlands and soils with lower oxygen that produce greater amounts of DOM, ultimately transported by increased runoff to rivers, lakes, and coastal oceans. This leads to higher concentrations of DOM and reduced UV transparency^35,39^. In regions such as the northeastern USA, extreme precipitation events have increased by as much as 71% between 1958 and 2012^1^. These extreme precipitation events can induce abrupt reductions in water clarity due to increased inputs of both dissolved and particulate substances to inland waters^3^. Extreme storm events contribute a major portion of the annual DOM flux from terrestrial to aquatic ecosystems. For example, a single hurricane event in 2011 (Irene) contributed 19% of the 2011 annual DOM exports from a forested catchment^40^. Space-for-time modeling based on data from over 1,000 boreal lakes in Norway indicate that future increases in air temperature and precipitation will increase DOM concentrations by as much as 65% in surface waters^41^. Modeling of carbon flux from the Mississippi River basin to the Gulf of Mexico from 2001–2010 showed that DOM export was approximately 30% higher during a wet year and 30% lower during a dry year compared to the average during the study period^4^. Modeling studies that focus on hydrologic changes and hold other factors such as land use change constant have estimated an increase in annual DOM export into the Gulf of Maine of up to 40–50% or more in some rivers from 1930 to 2013^42^. Most of this increase was during the fall and winter when sunlight for solar disinfection is low. The increase in DOM combined with seasonal decreases in incident solar radiation during the fall and winter (Fig. 1) will accentuate the reduction in SIP in coastal receiving waters during these periods. Indonesian rivers in peatland-dominated landscapes that drain into coastal oceans deliver twice the discharge volume and higher DOM concentrations during the wet season (35 mg L^−1^) compared to the dry season (28 mg L^−1^)^43^. Deforestation of Indonesian peatlands for agriculture may increase DOM by as much as 37% within the next 50 years in a worst-case scenario^44^.

**Figure 3:**
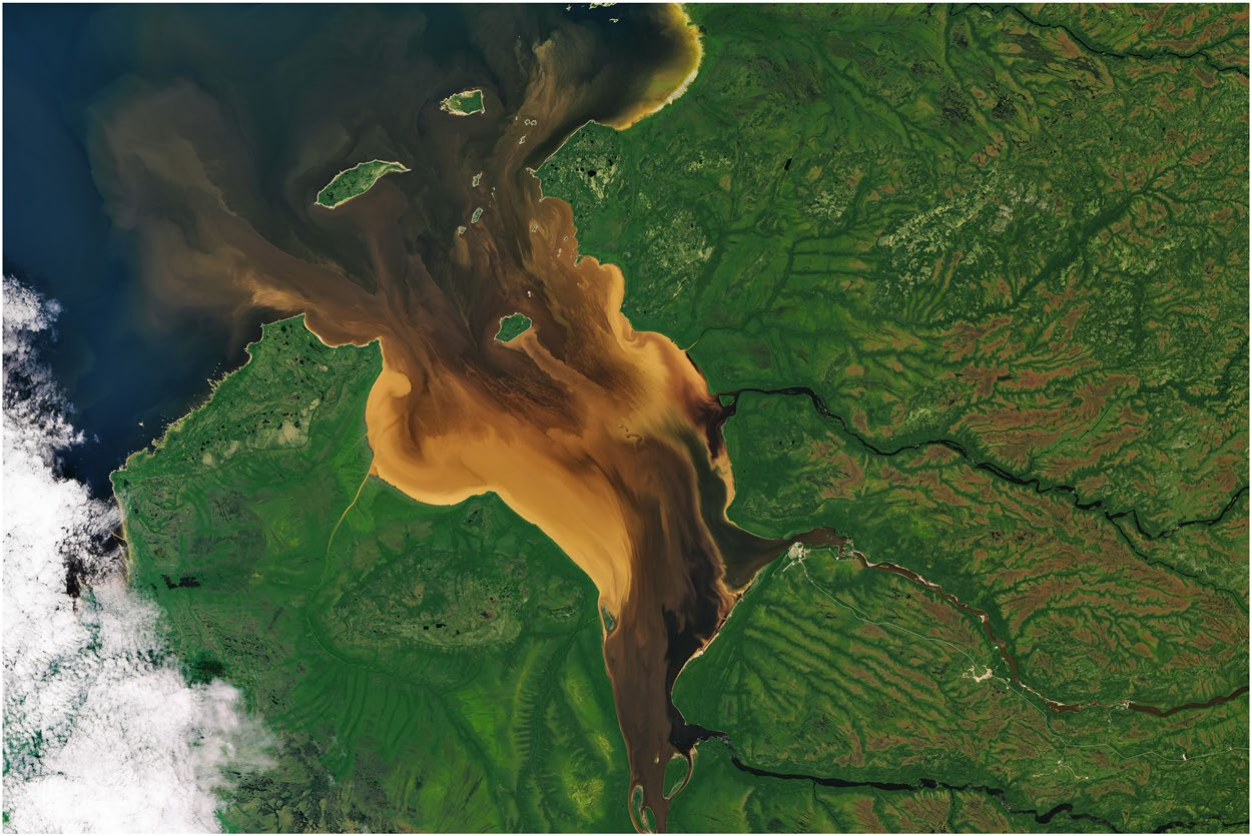
Rivers deliver substantial amounts of terrestrial DOM to coastal regions of lakes and oceans. Satellite photograph of rivers in Quebec transporting DOM and particulates through Rupert Bay into the southern end of the Hudson Bay, which reduces the SIP in these aquatic ecosystems. NASA Earth Observatory image by Joshua Stevens using USGS Landsat Data, downloaded from https://landsat.visibleearth.nasa.gov/view.php?id=88843.

Recent satellite imagery has enabled improved visualization of the input of terrestrially-derived DOM into the oceans in regions ranging from the Arctic to the sub-tropics^45,46^ (Fig. 3). Dilution, and processing of DOM by sunlight and microbes in estuaries and oceans, lead to a decrease in DOM with increasing distance from the shore^47^. This leads to a subsequently lower SIP and potential for greater pathogen survival in the nearshore vs. offshore regions (Figs 1 and 3). The tenfold greater abundance of zooplankton (copepod) parasites in nearshore vs. offshore oceanic environments is consistent with this spatial variation in SIP^48^.

While climate change is leading some regions to experience greater precipitation and heavier precipitation events, in regions such as the western USA, drought conditions have become prevalent in many recent years. Drought conditions increase water clarity and thus UV exposure in clear-water lakes^35^. Drought reduces the dissolved and particulate matter that is washed into inland and coastal waters, and increases water residence times, thus prolonging exposure to degradation of DOM by sunlight^35,47,49^. This greater water transparency may in turn lead to higher SIP in the affected surface waters. Adequate spectral absorbance and DOM data necessary to thoroughly explore the impact of warm, dry conditions on SIP are not available. However, two lines of evidence support an increase in SIP during warm, dry conditions such as drought. First, DOM output from rivers is lower in dry vs. wet seasons or years as discussed in the references above. Second, in the case study reported below, we show how the warm, dry summer periods are periods of increased water clarity and SIP.

## A case study of heavy rain reducing SIP

**Figure 4:**
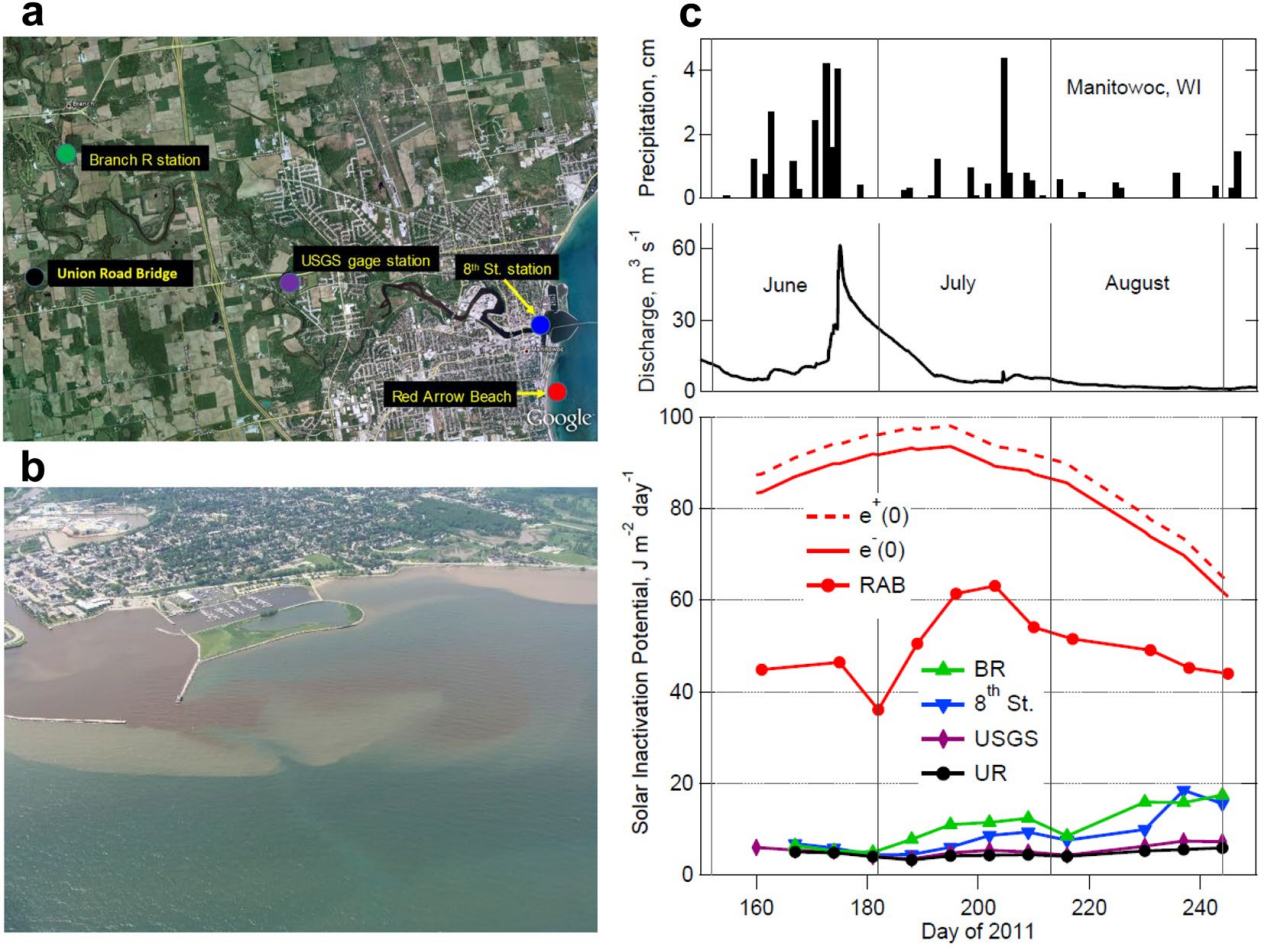
Effects of heavy rain on discharge and SIP in the Manitowoc River and Lake Michigan. (**a**) Map of sampling sites. (**b**) Plume of turbid, high DOM river water entering the lake following heavy rain events in late June 2011. (**c**) Precipitation, river discharge, and SIP averaged over the top 1 m depth at sites along the river and lake. SIP above (e+) and below (e−) lake surface and at Red Arrow Beach (RAB, a popular swimming site), 8^th^ St. (at bridge), USGS (gage station, discharge data source), BR (Branch River), UR (Union Road bridge). Map credit for (**a**): USDA Farm Service Agency, GeoEye, ©2010 Google Imagery ©2010. DigitalGlobe. Photo credit for (**b**): Curt Drumm.

**Figure 5:**
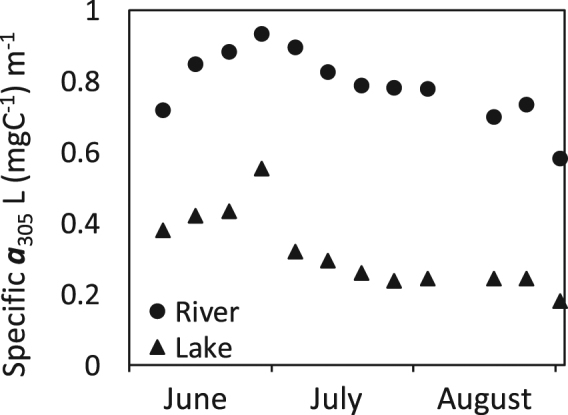
Changes in DOM darkness in the Manitowoc River and Lake Michigan during 2011. Darkness is expressed as DOC-specific absorbance at 305 nm. Note the strong increase in color that, along with increases in DOC concentration, contributed to the decrease in SIP during the period of rain in June before day 180. This was followed by a summer-long decrease in darkness due to decreases in terrestrial DOM inputs and photobleaching of the DOM by sunlight. Manitowoc River at the U.S. Geological Survey gage station and Lake Michigan, Red Arrow Beach.

Coastal and inland regions with high levels of disturbance from agriculture and other human activity may be particularly vulnerable to both increases in pathogens as well as increases in DOM following high precipitation events^49^. This in turn increases the threat of exposure to contaminated water and infectious diseases. In the Laurentian Great Lakes region the nexus of high human activity, heavy dependence on surface water resources, and climate change is creating substantial concerns for water quality and human pathogens^50^. This is illustrated here by a case study of the effects of heavy precipitation and runoff on river discharge, water transparency, and SIP where the Manitowoc River flows into Lake Michigan (Fig. 4). Following several smaller rain events in June, 2011, a heavy rain event on June 21 increased discharge from the Manitowoc River by an order of magnitude, releasing large inputs of darker, highly UV-absorbing terrestrial DOM into Lake Michigan in the vicinity of Red Arrow Beach, reducing the SIP by 22% (Figs 4 and 5). Then warmer temperatures and less precipitation in July reduced river discharge and DOM inputs as well as increased photobleaching, with a subsequent 75% increase in SIP from this low until another high precipitation event in late July. Because the input of pathogens increases in a manner similar to the input of DOM during heavy precipitation events^51^, the threat of infectious disease is compounded during these events, especially when rainfall coincides with abundance of the primary vectors of pathogens.

## DOM and SIP: A pathway to disease outbreaks

With globally increasing air temperatures, extreme precipitation events are increasing more rapidly than mean precipitation in many regions^1,52^. Precipitation-induced increases in runoff in turn enhance the introduction of pathogens to surface waters^51^. Our modelling suggests that increased DOM and reduced SIP will contribute to increased pathogen survival following heavy precipitation. In many cases, increases in infectious diseases are associated with lower water clarity as well as increased runoff, even in areas with advanced drinking water treatment^6^. For example, in the United States extreme precipitation events precede on the order of 68% of waterborne disease outbreaks^6^. Heavy precipitation has been implicated in cryptosporidiosis in Milwaukee, USA^53^, New Zealand^54^ and Spain^55^, as well as in emergency visits for diarrhea in the springtime in New York^56^. Recreational waters and harvesting of shellfish are often closed following extreme precipitation events due to the high probability of contamination^6^. As observed with many human waterborne diseases, amphibian chytridiomycosis tends to increase with increasing precipitation^57^, and decrease with drought^58^.

One of the few studies to directly examine the relationship between DOM, solar UV exposure, and epidemics of waterborne pathogens in nature involved *Metschnikowia bicuspidata*, a lethal fungal parasite of the important planktonic freshwater crustacean grazer *Daphnia* ^14^. Field incubation experiments that manipulated solar UV in two lakes revealed substantially higher infection prevalence in treatments shielded from natural solar radiation. In lakes with lower DOM concentrations, fungal epidemics started later in the year (when incident sunlight was reduced) and were smaller in magnitude.

## Caveats and a global context

**Figure 6:**
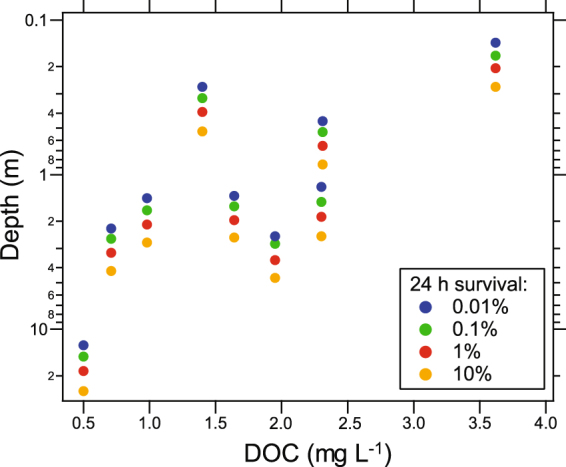
Changes in modeled depth of survival for the human pathogen *Cryptosporidium parvum* as a function of DOM concentration (as dissolved organic carbon, DOC). The 24 h survival is the percent inactivation in 24 h by solar UV-B near summer solstice. Data are from nine of the ten study lakes and sites in Fig. 1; Negra is missing due to the lack of DOM data. In the clearest, lowest DOM lakes high inactivation can occur even at depths of greater than 10 m, while at higher DOM concentrations inactivation depths decrease to a fraction of a meter. Note the variable response to DOM concentration which is due largely to variations in DOM quality (darkness, DOC-specific absorbance) among lakes. Less UV-sensitive pathogens will have correspondingly shallower inactivation depths, with the most UV-resistant species such as adenovirus not even being susceptible to solar UV inactivation at the surface of a lake within a single day.

The abundance of waterborne pathogens is regulated by multiple factors, and many of the environmental controls are system- and pathogen-specific. Several useful models that simulate the effects of natural sunlight and multiple other factors that control water-borne pathogens have been developed^8–10,15^, and additional modeling studies as well as data on DOM and UV absorbance spectra in natural surface waters^59^ are increasing our ability to quantitatively estimate how variations in DOM reduce solar UV disinfection. Our UV absorbance data from a wide variety of inland surface waters and modeling of SIP suggest that widespread reductions in water transparency are reducing the SIP for waterborne pathogens over space and time. In addition to DOM concentration, DOM quality (darkness) is critical to determining UV absorbance and thus SIP of pathogens. For example, if we use the absorbance spectra that are the basis for estimating SIP in Fig. 1 to plot the depth of different levels of solar inactivation of *Cryptospordium parvum* as a function of DOM concentration, we find an increasingly shallower depth for a given level of pathogen inactivation with increasing DOM concentration (as dissolved organic carbon, DOC, Fig. 6). These inactivation depths vary over two orders of magnitude due to differences in DOM concentration. The scatter apparent in this relationship is due to variation in the UV absorbing capacity (DOC-specific absorbance, or “darkness”) of the DOM among lakes, or even within lakes (Fig. 5). Variations in DOM quantity and quality are due largely to differences in hydrology, weather conditions, and climate in the given region^35^. Climate change is altering not only DOM quantity and quality, but also cloud cover in complex ways that will also alter SIP in surface waters.

Several factors may lead our model to underestimate SIP. First, we estimate SIP for only the top 1 meter of surface waters, while in very clear systems UV-B may penetrate deep enough for substantial SIP at depths deeper than 1 m (Fig. 6). This may lead to an underestimate of the effects of DOM on SIP. Second, the model uses only dissolved absorption coefficients, and yet there are many systems such as rivers and shallow reservoirs where inorganic particulates may account for a substantial portion of light attenuation in the water column. Third, nutrient pollution or climate warming may enhance algal growth, and blooms of filamentous algae such as *Cladophora* have been shown to shield human pathogenic bacteria from damaging UV, extending the viability of these pathogens for many days^60^. Fourth, our analysis is based on DNA damage by the UV-B component of the solar spectrum, but UV-A may also play an important role in solar inactivation of pathogens. While DOM attenuates UV-B most strongly, it also attenuates UV-A, which produces ROS such as singlet oxygen and hydrogen peroxide, that have been shown to inactivate pathogens due to damage to cell components other than DNA^10,19,30,61^. In some cases, the inactivation of viruses by sunlight may be greater in the presence of DOM than in its absence if the photoproducts increase virus inactivation more than the DOM reduces exposure to UV damage^10,61^. Species-specific UV tolerance and environmental factors such as pH, dissolved oxygen, and ionic composition of the water also need to be considered^10,32,61^. Oxygen depletion and pH may be particularly important in waste stabilization ponds where dissolved oxygen can be rapidly depleted and pH can exceed 8.5^32^. Warmer temperatures may also increase predation rates on pathogens, thus contributing to environmental inactivation. Though not present in some bacteriophages^62^, photoenzymatic repair of UV-damaged DNA is also dependent upon longer wavelength UV-A and visible light^63^, and may consequently reduce SIP, especially at sub-lethal levels of UV exposure. The presence or absence of photoenzymatic repair and variation in its spectral dependence among pathogen species as well as the attenuation of these longer wavelengths by DOM are also important processes that will influence SIP, and need to be explored further.

**Figure 7:**
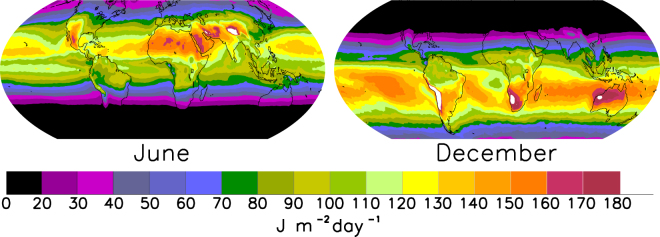
Global climatology of daily SIP at Earth’s surface. This was calculated with an atmospheric radiative transfer model using as input daily ozone column and climatological cloud data observed by satellites during 1990–2000. Computed spectral irradiances were weighted by the DNA action spectrum and integrated over 24 hours, to yield the daily doses that are used here as a proxy for SIP at the Earth’s surface (above the water surface). Maps were developed with IDL Version 8.4 (linux × 86_64 m64). (c) 2014, Exelis Visual Information Solutions, Inc. (http://www.harrisgeospatial.com/ProductsandTechnology/Software/IDL.aspx).

The importance of variation in SIP extends beyond inland and coastal ecosystems. SIP just above Earth’s surface can be estimated for any location and time given knowledge of the optical state of the atmosphere (mostly ozone and clouds) as well as season and latitude (Fig. 7). The reference SIP values from Table 1 (100 J m^−2^ day^−1^) are exceeded year-round only in the tropics, and in the summer only at latitudes <45°. Thus even in the most transparent waters SIP may be very low in higher latitude regions during the winter when sun angle is low, or at times when cloud cover is high. Current climate change scenarios project region-specific changes in cloud cover in the future^1,52^ that will affect UV exposure levels. For example, increases in cloud cover at high Northern latitudes will reduce incident UV^64^. However, for temperate latitudes, UV changes due to cloud changes are expected to be 5% or less by the end of this century^64^. At the global scale, recovery from ozone depletion is predicted to reduce incident UV by up to 40% in Antarctica, but will have smaller effects outside of the high southern latitudes^64^. In contrast, climate change induced melting of ice in the Arctic Sea may increase sub-surface exposure to UV by tenfold, and improvements in air quality over populated areas may decrease aerosols and potentially increase incident UV by 10–20% or more^64^. These changes, along with those affecting water transparency and runoff, will alter the potential for solar UV disinfection of waterborne pathogens in the coming decades. The disproportionally greater increase in human populations in coastal regions (e.g. 39% growth in coastal regions vs. 13% for the overall population in the USA since 1970^51^) as well as heavy use of inland waters for waste disposal will amplify the potential exposure of humans to contaminated waters. Livestock and wildlife that depend on surface waters are also likely to experience an increase in exposure to waterborne pathogens and infectious diseases, further threatening the ecosystem goods and services provided by inland and coastal surface waters.

## Conclusions

The spectral absorbance data on natural water bodies and modeling of SIP presented here extend previous studies by demonstrating the potential for precipitation to elevate DOM concentrations in inland and coastal waters, thus decreasing the potential for solar UV radiation to inactivate pathogens. This study is a first step and further empirical surveys and experimental studies are needed to confirm the importance of solar UV inactivation relative to other environmental controls of pathogen abundance. Environmental data collected under natural solar radiation conditions that include spectral attenuation in the water column across environmental gradients of temperature, pH, and other important regulators of pathogen survival are necessary to identify the complex interactions that will ultimately regulate pathogen abundance in surface waters. Studies of species-specific variations in pathogen susceptibility to solar UV damage as well as repair mechanisms will also be essential to estimating the importance of SIP relative to other environmental controls of waterborne diseases of humans and wildlife.

## Methods

### Modeling the Solar Inactivation Potential (SIP)

The ultraviolet irradiance reaching Earth’s surface was calculated with the Tropospheric Ultraviolet-Visible (TUV, version 5.3) model^34^. The model propagates the incident solar spectral irradiance^65^ through the atmosphere^66^, accounting for absorption and scattering by O_3_ and air molecules. Separate outputs were obtained for the direct solar beam and diffuse (skylight) irradiances, from 280 to 400 nm in 1 nm steps.

In-water radiation was computed from the incident atmospheric radiation by applying Snell refraction and Fresnel reflection (for unpolarized light) at the air water interface, and Beer-Lambert propagation to depths below the surface. For the direct beam, the entry and propagation geometry is straightforward because the angle of incidence at the air-water interface equals the solar zenith angle. Diffuse radiation (which frequently exceeds the direct solar beam at UV wavelengths) was assumed to be hemispherically isotropic, and was subdivided into N angular sectors of equal area measured from the zenith (cosine-weighted), so that each of the N beamlets had its own angle of incidence in air, and then (through Snell’s law) its own angle under water. The total irradiance at any depth was computed by summing the contributions from the direct beam and the N diffuse beamlets, each component having experienced a slightly different propagation angle and thus slightly different Beer-Lambert attenuation. Typically, N = 10 was used, with no significant improvement from finer subdivisions.

While the atmospheric part of the model includes both absorption and scattering in all directions, the under-water extension describes only absorption, not scattering. We specifically neglect scattering by suspended particles and reflection from the bottom. Consequently, no upward-propagating radiation is considered within the body of water, avoiding ambiguities related to scattering phase functions, angular radiance distributions, and internal reflections near the critical angle. This simplification is justified by noting that *in-situ* measurements of attenuation coefficients (which include both absorption and scattering) are frequently in excellent agreement with measurements on filtered samples (absorption only), demonstrating the predominance of absorption^67^. Absorption spectra of water samples obtained from different lakes were applied to the Beer-Lambert absorption in TUV, as described above, to estimate the spectral irradiance *F*(λ,*z*, *t*) as a function of wavelength λ, depth *z*, and time *t*. The biologically effective dose rate for a particular endpoint is then obtained by weighing the spectral irradiance with the biological sensitivity (action) spectrum for that endpoint, *B*(λ):

1 $$Dose\,Rate=\int F(\lambda ,z,t)\,B(\lambda )\,{\rm{d}}\,\lambda $$

Here, we use the action spectrum for DNA damage^68^, since there is extensive evidence that the DNA molecule is a primary target for inactivation of viruses^33^. Other cellular constituents could clearly be influenced too including capsid proteins in viruses^33^. The action spectrum was re-normalized at 254 nm to facilitate comparison of our estimates of ambient solar exposures to those from laboratory studies using Hg lamps at this wavelength. We define the Solar Inactivation Potential (SIP) as the daily dose of DNA-weighted UV radiation,

2 $${\rm{SIP}}=\iiint F({\rm{\lambda }},z,t)\,B({\rm{\lambda }}){\rm{d}}\,{\rm{\lambda }}\,{\rm{d}}z\,dt/{\rm{\Delta }}t$$

where Δ*t* = 1 day.

The global climatology of the daily SIP (Fig. 7) was calculated with the TUV model and satellite-based observations (1990–2000) of total ozone and clouds, as previously described by Lee-Taylor *et al*.^69^ but modified to use the Setlow DNA action spectrum normalized at 254 nm. The TUV model version 5.3 used in this study is available openly and freely at: https://www2.acom.ucar.edu/modeling/tropospheric-ultraviolet-and-visible-tuv-radiation-model.

### Measuring DOM and UV transparency in surface waters

For the data presented in Figs 1 and 2 the methods for measuring DOM (as DOC, mg CL^−1^), spectral absorption coefficients (m^−1^), and UV transparency (1% depths estimated from diffuse attenuation coefficients based on subsurface measurements only) followed previously established methods^39,70^. Water samples were collected in the surface waters of lakes with a Van Dorn bottle and spectral scans carried out on GF/F-filtered subsamples in a Shimadzu Total Organic Carbon Analyzer (TOC-5000 or TOC-V_CPH_) corrected with Milli-Q deionized water blanks and calibrated with a diluted, certified DOC standard (Aqua Solutions, 50 mg L^−1^ potassium biphthalate). Dissolved absorbance was obtained from GF/F-filtered samples with a Shimadzu UV-Visible spectrophotometer (UV-160U, UV-1601, or UV-1650). Raw absorbance values were corrected by subtracting the average absorbance of a Milli-Q water blank between 775–800 nm and calculating Naperian dissolved absorption coefficients^70^. Water transparency to 320 nm UV was measured with submersible, profiling radiometers (PUV, or BIC, Biospherical Instruments, Inc., San Diego, CA).

For the data presented in Figs 4 and 5, water samples were collected from the Manitowoc River at Union Rd (44°6′42.206″N, 87°46′51.938″W), at Michigan Ave. (44°6′21.712″N, 87°42′57.744″W), and at the 8^th^ St. Bridge in Manitowoc WI (44°5′31.776″N, 87°39′27.583″W), at the Branch River at Union (44°8′5.010″N, 87°45′55.181″W), and Lake Michigan at Red Arrow Beach on Lake Michigan (44°4′34.000″N, 87°39′19.782″W). (Fig. 4a) Red Arrow beach was sampled on Sundays, Mondays, Fridays and Saturdays from June 3, 2011 to September 5, 2011. The four river sites were sampled from June 4, 2011 to September 1, 2011. At three of the river sites, each sample used for our measurements was a composite of six subsamples (each 290 ml) taken from the stream’s thalweg using a Niskin sampler. At the 8^th^ St. Bridge Manitowoc River site (river mouth), twenty sub-samples (each 75 ml) were composited to create one 1800 ml sample. All samples were prepared for UV-visible, and Total Organic Carbon (TOC), measurements within 6 hours of collection. At Red Arrow beach, water samples were obtained from Lake Michigan for UV-visible spectral measurements and DOC analysis using Total Organic Carbon (TOC) certified amber glass bottles with Teflon lined caps (Scientific Products) at depths that were 0.15–0.20 m below the water’s surface in water that was 0.5 m deep. Other water samples for microbial analysis were collected as described in Section 9060 of Standard Methods for the Examination of Water and Wastewater (1999) 0.15–0.20 m below the water’s surface in water that was 0.5 m deep, using sterile polyethylene terephthalate bottles (Idexx).

These Manitowoc region water samples were prepared for UV/visible spectral measurements and dissolved organic carbon (DOC) analysis by filtering each water sample through a 0.2 µm polycarbonate Millipore filter within 6 hours of collection. The filtrates were stored in TOC approved bottles at 4 °C until analysis. UV-visible absorption spectra of the water samples were determined using a Perkin-Elmer LAMBDA^TM^ 35 UV/Visible Spectrophotometer. Measured absorbances (A_λ_) in the UV-visible spectra were used to calculate absorption coefficients (i.e., *a*_λ_ = 2.303(A_λ_/*L*) where *L* is the path length in m.

During every sampling activity in the Manitowoc region, physical and biochemical characteristics of the water were also measured using a YSI sonde, Model 6600 M, including water temperature, pH, specific conductivity, dissolved oxygen, chlorophyll and turbidity. Water quality and current condition data were also recorded by the US Geological Survey at the gage station site at Michigan Avenue on the Manitowoc River (https://waterdata.usgs.gov/usa/nwis/uv?site_no=04085427). Precipitation and discharge data were obtained from the USGS gaging station at the 8^th^ Street Bridge in Manitowoc, WI (Fig. 4a).

### Data availability

The data from this study are available from the authors on reasonable request. See author contributions for specific data sets.
